# Data on the alizarin red S adsorption from aqueous solutions on PAC, treated PAC, and PAC/γ≈Fe_2_O_3_

**DOI:** 10.1016/j.dib.2018.08.170

**Published:** 2018-08-31

**Authors:** Bahram Kamarehie, Ali Jafari, Mansour Ghaderpoori, Mohammad Amin Karami, Khadijeh Mousavi, Afshin Ghaderpoury

**Affiliations:** a Department of Environmental Health Engineering, School of Health and Nutrition, Lorestan University of Medical Sciences, Khorramabad, Iran; b Nutritional Health Research Center, Lorestan University of Medical Sciences, Khorramabad, Iran; c Student Research Committee, Shahid Beheshti University of Medical Sciences, Tehran, Iran

**Keywords:** Adsorption, Textile effluents, Dye, Aqueous solutions, Fe_2_O_3_

## Abstract

Three types of adsorbents of powdered activated carbon (PAC), treated PAC, and PAC/γ ≈ Fe_2_O_3_ nanocomposite were used. The adsorption experiments were performed in batch conditions. pH_ZPC_ of PAC/γ ≈ Fe_2_O_3_ was 6.7. As a result, at lower than pH_ZPC_, acidic pH, the adsorption of alizarin red S on PAC/γ ≈ Fe_2_O_3_ was favourable. The maximum of alizarin red S adsorption of PAC, treated PAC, and PAC/γ ≈ Fe_2_O_3_ was 24.5 mg/g, 57.8 mg/g, and 112.56 mg/g, respectively. The models of Langmuir and pseudo-first-order were a fit model to describe the adsorption isotherm and the Kinetic, respectively. The PAC/γ ≈ Fe_2_O_3_ is a promising class of the adsorbents in the adsorption of various dyes from textile effluents.

**Specifications Table**

**Table:** 

Subject area	*Wastewater treatment*
More specific subject area	*Adsorption*
Type of data	*Table, figure*
How data was acquired	*Spectrophotometer RD-5000(UV-UVIS, 570 nm)*
Data format	*Analyzed,*
Experimental factors	*The adsorption experiments were performed in batch conditions. The main variables studied were initial dye concentration, pH, reaction time, and treated PAC and PAC/γ ≈ Fe*_*2*_*O*_*3*_*dosage. An adsorbent of PAC, treated PAC and PAC-γ-Fe*_*2*_*O*_*3*_*nanocomposite was added to 100 mL of alizarin red S solution. The residual dye was measured by a spectrophotometer DR-5000 (UV-UVIS, 350 nm).*
Experimental features	*In the first step, in order to prepare treated PAC. After separation, the dark-brown precipitate was washed several times with methanol to remove the residual matter.*
Data source location	*Khorramabad, Lorestan University of Medical Sciences, Iran (lums.ac.ir)*
Data accessibility	*Data are included in this article*
Related research article	*S. Golmohammadi, M. Ahmadpour, A. Mohammadi, A. Alinejad, N. Mirzaei, M. Ghaderpoori, A. Ghaderpoori. Removal of blue cat 41 dye from aqueous solutions with ZnO nanoparticles in combination with US and US-H2O2 advanced oxidation processes. Environmental Health Engineering and Management Journal. 3 (2016) 107-13*

**Value of the data**

● The data from the present study showed that the modification of conventional absorbents can be used to considerably enhance the ability to remove environmental pollutants.
● The data obtained can be used to complete the information in literature on the removal of dye compounds from water environments and industrial effluents.

## 1 Data

**Fig. 1:**
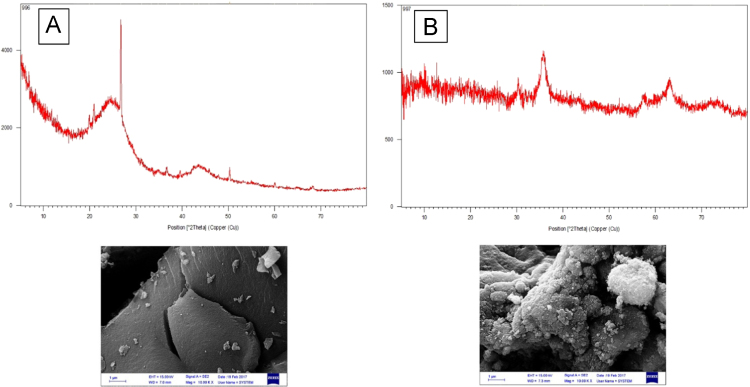
The XRD pattern and SEM images of treated PAC (A) and PAC/γ-Fe_2_O_3_ nanocomposite (B).

**Fig. 2:**
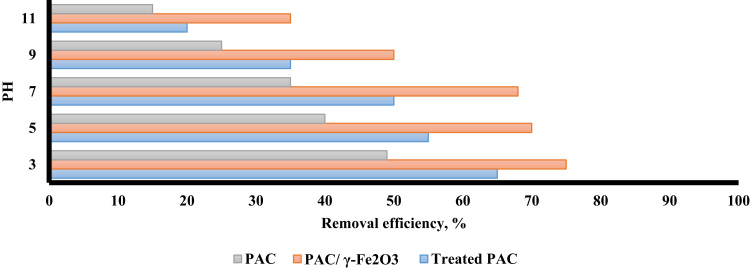
The effect of solution pH on alizarin red S adsorption by PAC, treated PAC, and PAC/γ-Fe_2_O_3_ nanocomposite.

**Fig. 3:**
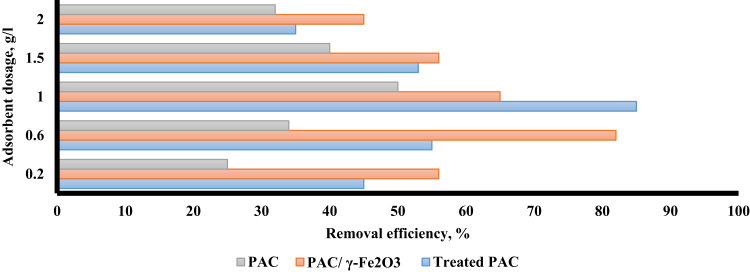
The effect of adsorbent dose of PAC, treated PAC, and PAC/γ-Fe_2_O_3_ nanocomposite on alizarin red S adsorption.

**Fig. 4:**
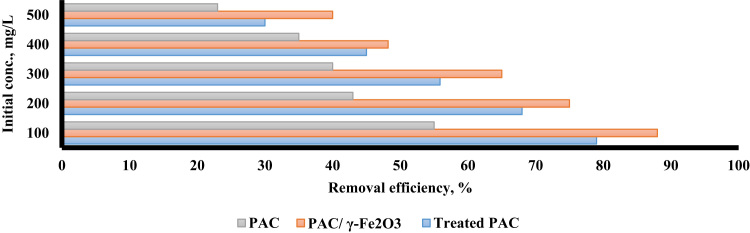
The effect of initial concentration of dye on alizarin red S adsorption by PAC, treated PAC, and PAC/γ-Fe_2_O_3_ nanocomposite.

The XRD pattern and SEM for treated PAC and PAC/γ ≈ Fe_2_O_3_ nanocomposite are presented in Fig. 1. Based on BET, the surface area of PAC, treated PAC, and PAC/γ ≈ Fe_2_O_3_ were found to be 389 m^2^/g, 550 m^2^/g, and 400 m^2^/g, respectively. Fig. 2 shows the effect of solution pH of PAC, treated, PAC and PAC/γ ≈ Fe_2_O_3_ nanocomposite on alizarin red S adsorption. The results of the study showed that the pH of the zero point (pH_ZPC_) was 6.5. Fig. 3 shows the effect of adsorbent dose of PAC, treated PAC, and PAC/γ ≈ Fe_2_O_3_ nanocomposite on alizarin red S adsorption. Fig. 4 depicts the effect of initial concentration of PAC, treated PAC, and PAC/γ ≈ Fe_2_O_3_ nanocomposite on alizarin red S adsorption. The constants of isotherm models for PAC, treated PAC and PAC/ γ-Fe_2_O_3_ nanocomposite on alizarin red S adsorption are given in Table 1. As illustrated in Table 1, the isotherm model of Langmuir for PAC, treated PAC and PAC/γ-Fe_2_O_3_ nanocomposite has the highest *R*^2^ (e.g. square correlation). Therefore, this model was the most suitable model to express alizarin red S adsorption onto the adsorbents. Also, the constants of kinetics models for PAC, treated PAC and PAC/γ-Fe_2_O_3_ nanocomposite on alizarin red S adsorption are summarized in Table 2. As illustrated in Table 2, the kinetic model of pseudo-second order for PAC, treated PAC and PAC/γ-Fe_2_O_3_ nanocomposite has the highest *R*^2^. As a result, this model was the most suitable kinetics model for alizarin red S adsorption onto the prepared adsorbents.

**Table 1:** The constants of isotherm models for alizarin red S adsorption by PAC, treated PAC, and PAC/γ-Fe_2_O_3_ nanocomposite.

		PAC	Treated PAC	PAC/γ ≈ Fe_2_O_3_
Langmuir	*q*_max_	24.5	57.8	112.56
	KL	1.05	1.66	2.45
	*R*^2^	0.88	0.89	0.89
Freundlich	*K_F_*	1.43	1.68	2.04
	*n*	1.12	1.78	2.6
	*R*^2^	0.64	0.77	0.87

**Table 2:** The constants of kinetics models for alizarin red S adsorption by PAC, treated PAC, and PAC/γ-Fe_2_O_3_ nanocomposite.

			PAC		Treated PAC		PAC/γ ≈ Fe_2_O_3_	
			100 mg/l	200 mg/l	100 mg/l	200 mg/l	100 mg/l	200 mg/l
Psudo-first-order		*K*_1_	0.0164	0.0136	0.0234	0.0289	0.0235	0.0267
		*R*^2^	0.845	0.876	0.985	0.896	0.976	0.979
		*q*_cal_	22.54	28.92	39.63	47.16	61.45	90.20
Psudo-second-order		*K*_2_	0.0198	0.0204	0.0342	0.0356	0.0435	0.0567
		*R*^2^	0.845	0.876	0.985	0.896	0.976	0.979
		*q_m_*	24.36	28.41	41.45	50.45	68.95	89.78

## 2 Experimental design, materials, and methods

### 2.1 Materials

The chemicals including hydrochloric, hydrochloric, powered activated carbon (PAC), iron chloride tetrahydrate, iron chloride tetrahydrate, and alizarin red S were used. These high purity chemicals were purchased from Merck and Sigma-Aldrich.

### 2.2 Preparation of treated PAC and PAC-γ-Fe_2_O_3_ nanocomposite

For PAC coatings by γ ≈ Fe_2_O_3_, the methodology of previous studies were obeyed [1], [2]. In the first step, in order to prepare treated PAC, this method was as follows: 20 g of PAC was added to a solution of 5 M nitric acid (Approximately 150 mL). The solution was placed at 70 ° C for 1 h. In the next step, in order to prepare activated carbon coated with γ-Fe_2_O_3_, this method was as follows: treated PAC [4.2 g], FeCl_3_–6H_2_O [21.6 g, purity > 99%], and FeCl_2_–4H_2_O [8 g, purity > 98%], were added to a solution of 2 M hydrochloric (Approximately 100 mL, purity 37%). The NH_3_. H_2_O solution a solution of 2 M NH_3_. H_2_O solution (Approximately 300 mL) was added to the previous solution for 2 h. Finally, the remaining precipitate was separated by a magnet. After separation, the dark-brown precipitate was washed several times with methanol to remove the residuals. After washing, the final product was dried at 70 °C for 24 h. After preparation of treated PAC and PAC/γ ≈ Fe_2_O_3_ nanocomposite, their characterizations were determined using SEM, XRD, and BET techniques [1], [2], [3], [4], [5], [6], [7], [8], [9], [10], [11], [12], [13], [14], [15], [16].

### 2.3 The adsorption experiments

The adsorption feasibility of Alizarin red S was studied by PAC, treated PAC, and PAC/γ ≈ Fe_2_O_3_ nanocomposite. The adsorption experiments were performed in batch conditions. The main variables studied were initial dye concentration, pH, reaction time, and treated PAC and PAC-γ-Fe_2_O_3_ dosage. At the first step, a stock Alizarin red S solution (C_14_H_8_O_4_, 1000 mg/l, 240.21 g/mol, *pK_a_* = 6.9) was prepared and stored under standard conditions. An adsorbent of PAC, treated PAC and PAC-γ-Fe_2_O_3_ nanocomposite was added to 100 mL of Alizarin red S solution. Equation C_1_V_1_ = C_2_V_2_ was used to prepare different concentrations of stock solution. The solutions of 0.1 N HCl and NaOH were used to adjust the desired pH. The residual dye was measured by a spectrophotometer DR-5000 (UV-UVIS, 350 nm) [3].
